# Time-Resolved FRET -Based Approach for Antibody Detection – A New Serodiagnostic Concept

**DOI:** 10.1371/journal.pone.0062739

**Published:** 2013-05-07

**Authors:** Satu Saraheimo, Jussi Hepojoki, Visa Nurmi, Anne Lahtinen, Ilkka Hemmilä, Antti Vaheri, Olli Vapalahti, Klaus Hedman

**Affiliations:** 1 Department of Virology, Infection Biology Research Program, Haartman Institute, University of Helsinki, Helsinki, Finland; 2 BN Product & Services, Finland Laboratory Division, Turku, Finland; 3 Helsinki University Central Hospital Laboratory Division, Helsinki, Finland; 4 Department of Veterinary Biosciences, University of Helsinki, Helsinki, Finland; Tulane University, United States of America

## Abstract

Förster resonance energy transfer (FRET) is a phenomenon widely utilized in biomedical research of macromolecular interactions. In FRET energy is transferred between two fluorophores, the donor and the acceptor. Herein we describe a novel approach utilizing time-resolved FRET (TR-FRET) for the detection of antibodies not only in a solution-phase homogenous assay but also in single- and two-step solid-phase assays. Our method is based on the principle that the Y-shaped immunoglobulin G molecule is able to simultaneously bind two identical antigen molecules. Hence, if a specific IgG is mixed with donor- and acceptor-labeled antigens, the binding of antigens can be measured by TR-FRET. Using donor- and acceptor-labeled streptavidins (SAs) in conjunction with a polyclonal and a monoclonal anti-SA antibody we demonstrate that this approach is fully functional. In addition we characterize the immune complexes responsible for the TR-FRET signal using density gradient ultracentrifugation and solid-phase immunoassays. The homogenous TR-FRET assay described provides a rapid and robust tool for antibody detection, with a wide potential in medical diagnostics.

## Introduction

Förster resonance energy transfer (FRET) is a process in which a donor molecule in excited state transfers its excitation energy through dipole-dipole coupling [1] to an acceptor fluorophore, when the two are brought into close proximity (typically less than 10 nm) [2], [3]. Upon excitation at a characteristic wavelength the energy absorbed by the donor is transferred to the acceptor, which in turn emits the energy. The level of light emitted from the acceptor fluorophore is proportional to the degree of donor-acceptor complex formation [4]. FRET between chromophores is characterized by Förster radius (R_0_), the distance at which the efficiency of FRET is 50% [5]. This phenomenon has been applied, among others, to study of protein-protein interactions, antigen-antibody binding, ligand-receptor interactions, DNA hybridization and DNA-protein binding [2], [6]–[11].

Many molecules present in biological fluids such as serum are naturally fluorescent, thus limiting the use of conventional fluorophores. The interfering autofluorescence can be minimized by utilizing long-lived fluorophores and time-resolved (TR; delay between excitation and emission) detection. Time-resolved fluorometry (TRF) relies on the long fluorescence emission half-life of lanthanides, rare earth elements such as europium (Eu) and terbium (Tb) [12]–[16]. TR-FRET unites the TRF and FRET principles. In TR-FRET, chelated or cryptic lanthanides are typically used as donors for acceptors such as Alexa Fluors™ or cyanine dyes (e.g. Cy5).

Most serodiagnostic assays, such as enzyme-linked immunosorbent assay (ELISA), are heterogeneous approaches with several steps of washing and reagent incubations. Such methods tend to consume time, labour and cost. To overcome such issues, TR-FRET-based homogeneous assays have been employed widely in research and diagnosis to detect e.g., antibodies, disease markers and receptor-ligand binding. [17]–[20].

The immunoglobulin G (IgG) molecule by nature is bivalent, that is, each IgG molecule can bind two identical antigens through its Fab arms. The hinge regions between the Fab and Fc parts are relatively flexible (with slight differences between different IgG subclasses) allowing the movement of the two Fab arms towards and away from each other [21]–[23]. Due to the flexibility of IgG molecule the antigens bound to its Fab arms can be brought into close proximity to each other. We reasoned that if one of the antigens is labeled with a donor fluorophore and the other with an acceptor fluorophore, the antigen binding (in the 50% cases forming a heterologous donor-acceptor pair) could be measured utilizing TR-FRET. Since TR-FRET minimizes background autofluorescence, this approach should enable the detection of specific antibodies directly from biological fluids such as serum or even a blood droplet.

In this report we provide proof-of-principle for a TR-FRET based homogenous immunoassay using donor- and acceptor-coupled streptavidins (SAs) as a model system in conjunction with polyclonal and monoclonal anti-SA antibodies at various concentrations. As the chosen antigen is tetrameric by nature, we were also interested in studying the molecular determinants of the FRET-inducing complexes. Therefore we characterized the antigen-antibody complexes accounting for the TR-FRET signal using density gradient ultracentrifugation.

## Materials and Methods

### Proteins and Antibodies

Alexa Fluor™ 647 (AF647) and europium-W1024-chelate -labeled SA (AF647-SA and Eu-SA) used as antigens in our assays were ordered from Invitrogen and PerkinElmer, respectively. Monoclonal antibody (MAb) against SA (mouse IgG2, clone S3E11, 6.1 mg/ml) was from Thermo Scientific (Pierce Protein Biology Products). IgG fractions of rabbit anti-SA sera (from Abcam Ltd 1 mg/ml and Springbioscience Inc 0.2 mg/ml) were used in our assays as representatives of polyclonal antibodies (PAbs). The antibody from Springbioscience was used only when determining the sizes of the immune complexes. IgG fraction of rabbit antiserum against glutathione-S-transferase (GST, Millipore, 0.5 mg/ml) was used as a control for the anti-SA antibodies. Horseradish peroxidase (HRP) labeled secondary antibodies, polyclonal swine anti-rabbit IgG and polyclonal rabbit anti-mouse IgG were from Dakocytomation (Agilent Technologies). Protein A (2 mg/ml) was from GE Healthcare and bovine serum albumin (BSA) from Sigma-Aldrich.

#### Generation of anti-SA Fab fragments

The anti-SA MAb (S3E11) was fragmented using papain. Papain 25 mg/ml, 40 U/mg, (P3125, Sigma-Aldrich) was diluted 1 to 10 in 100 mM L-cysteine (in PBS, pH 7.4) and preactivated by 15 min incubation at RT. Anti-SA MAb was diluted 1 to 3 in reaction buffer (10 mM L-cysteine and 10 mM EDTA in PBS) yielding a 2 mg/ml antibody solution. Preactivated papain was further diluted 1 to 100 in reaction buffer. The cleavage reaction was set up by mixing 1 part of papain solution with 1 part of diluted antibody. The cleavage was carried out at 37°C for 4 h, after which papain was inactivated by addition of 0.3 M iodoacetamide (in PBS) to reach final concentration of 30 mM.

Adsorption with Gammabind™ Plus Sepharose™ (GE Healthcare) was used to remove intact IgG molecules and to roughly separate the cleavage products (Fab and Fc parts) from each other. Briefly, Gammabind™ beads were equilibrated to PBS+(PBS with additional 150 mM NaCl and 0.01% of Tween 20), mixed with the reaction mixture and incubated 15 min at RT. The beads were sedimented by centrifugation (500×g, 2 min) and the supernatant recovered. The beads were washed twice with 150 µl of PBS+and the resulting supernatants were pooled together with the initial supernatant. The supernatant pool containing the Fab fragments and inactivated papain was concentrated and the buffer exchanged to PBS using Amicon Ultra 10 kDa centrifugal filter units (Millipore) according to product instructions. After buffer exchange the protein concentration was quantified using BCA Protein Assay kit (Pierce) according to manufacturer’s instructions. The final protein concentration of Fab containing fraction was 0.65 mg/ml. The success of cleavage and purification was analyzed by separating an aliquot of Gammabind™ beads and concentrated Fab fragments on non-reducing 8% SDS-PAGE. The proteins were stained with Coomassie Brilliant Blue and detected by scanning the gel at 700 nm in Odyssey Infrared Imaging System.

### Enzyme-linked Immunosorbent Assay (ELISA)

A twofold dilution series (from 1/400 to 1/409 600, corresponding to 17 nM to 0.016 nM) of both anti-SA PAb and MAb (initial IgG concentration for both 1 mg/ml, roughly corresponding to 6.6 µM) was done in Tris-buffered saline (TBS, 50 mM Tris-HCl pH 7.4 and 150 mM NaCl) containing 0.2% BSA (TBS-BSA). 100 µl of each dilution was pipetted into wells of SA coated microwell strips (96-well format, BioBind assembly, Thermo Electron Oy) followed by one hour incubation at 37°C. TBS-BSA was used as a negative control. The wells were washed four times with 200 µl of TBS, 100 µl/well secondary antibody added (anti-rabbit-HRP and anti-mouse-HRP, both diluted 1∶500 in TBS-BSA) followed by 1 hour incubation at 37°C. After four washes with TBS (200 µl each) 100 µl of HRP substrate (Dakocytomation) was added and the plate was incubated 20 min at RT. The reaction was terminated by addition of 0.5 M H_2_SO_4_ (100 µl/well) and the results were read (absorbance at 492 nm). This experiment was repeated in total three times with similar results.

SA-ELISA was also used to test the ability of the Fab fragments, cleaved from mouse anti-SA antibody, to bind SA. Briefly, a dilution series (1∶100–1∶6400) of the Fab solution and intact anti-SA MAb (40 nM to 0.625 nM) were pipetted on wells of streptavidin-coated 96-well plates (BioBind assembly, Thermo Electron Oy) in duplicate. After 45 min incubation at 37°C the plate was washed three times with PBS-T (PBS+0.05% Tween 20) and 1 to 1000 diluted HRP-labeled rabbit anti-mouse antibody was added followed by 45 min incubation at 37°C. The plate was then washed three times with PBS-T followed by addition of TMB substrate solution (Sigma-Aldrich). The reaction was stopped by addition of 50 µl 0.5 M H_2_SO_4_ and the results were read at 450 nm. The experiment was performed in duplicate.

### TR-FRET Assays

After optimization, the basic protocol was: Eu-SA, AF647-SA and antibodies were diluted in TBS-BSA. Throughout this report, the concentrations of Eu-SA and AF647-SA are given separately (total SA concentration is the sum of the two). All experiments were performed in duplicate and repeated several times; representative results are shown. TR-FRET values were measured with Wallac Victor^2^ fluorometer (PerkinElmer) by excitation at 320 nm followed by a delay of 70 µs before recording fluorescent counts for 100 µs with 615 nm (Eu) and 665 nm (AF647) emission filters. To take into account the emission of Eu at 665 nm the measured TR-FRET values were normalized according to the following equation: AF647_N_ = AF647–k*Eu, where AF647_N_ = normalized AF647 fluorescent counts, AF647 = unnormalized A647 counts (at 655 nm), k = Eu emission at 665 nm/Eu emission at 615 nm and Eu = Eu fluorescent counts (at 615 nm). The constant k was found to be independent of Eu-SA concentration whereby a value of 0.001342 (average of AF647- to Eu-counts in Eu-SA dilutions of 1∶1000 to 1∶8000) was used in subsequent calculations. With reactions having low AF647 counts and high Eu counts, the normalization would occasionally result in a negative value; in these cases we used the buffer background (typically 3 to 10 counts) as the final value.

#### Solution-phase homogenous assay

In the solution-phase TR-FRET assays, the final reaction mixture (20 µl) consisted of 10 µl of the antigen mix and 10 µl of antibody solution that were dispensed onto a 384-well microplate. The suitability of two microplates (ProxiPlate-384 Plus F, Black 384-shallow well Microplate, and OptiPlate-384, White Opaque 384-well Microplate, both from PerkinElmer) was tested. Irrespective of the plate type similar results were obtained; thus to minimize reagent use in the assays, we chose the Black Proxiplate due to its smaller well size.

To explore the prerequisites of FRET-pair formation with the chosen antigens and antibodies, we cross-titrated the antigens at concentrations of 0.5 nM, 1.0 nM, 2.0 nM, 4.0 nM and 8.0 nM and antibodies at concentrations of 66.6 nM, 33.33 nM, 16.7 nM, 8.5 nM and 4.16 nM for the anti-SA MAb and PAb, and 33.33 nM, 16.7 nM, 8.5 nM, 4.16 nM and 2.08 nM for the anti-GST PAb. To test the optimal ratio of the labeled antigens we kept the Eu-SA concentration constant at 4 nM and varied the AF647-SA concentration in the range of 16 nM, 8 nM, 4 nM, 2 nM and 1 nM separately with PAbs and MAb. Additionally, we studied the effect of reaction time on the TR-FRET responses by measuring the plates after incubation at 4°C or RT for 15 min to 24 h. The experiments were performed in duplicate and repeated several times with similar results.

To test the effect of dissociating agents sodium dodecyl sulfate (SDS) and urea on formation of TR-FRET positive immune complexes, we diluted the antigens and IgG (anti-SA PAb) in TBS-BSA containing these agents at various concentrations (2 M, 1 M, 0.5 M and 0.25 M for urea and 0.5%, 0.25%, 0.1% and 0.05% for SDS) prior to dispensing onto 384-well plate. The antigen concentration was kept constant (4 nM Eu-SA and 4 nM AF647-SA) while the antibody was diluted serially (20 nM, 10 nM and 5 nM of total IgG). The experiments were performed in duplicate and repeated several times, representative results shown.

The ability of Fab fragments to induce TR-FRET was tested by mixing various concentrations (120 nM to 7.5 nM) of Fab fragments with Eu-SA and AF647-SA (both 8 nM).

#### Solid-phase TR-FRET assays

Black Optiplate-384 F HB (Perkin Elmer) microplate was coated with protein A at 2 µg/µl dilution in TBS (optimized by titration) overnight at room temperature (RT), blocked three times 10 minutes with TBS-BSA. Washes were performed four times with TBS. Subsequent reactions occurred in TBS-BSA at 37°C.

In single-step assays the final reaction mixture (40 µl) consisted of 20 µl of antigen mix and 20 µl of polyclonal anti-SA dilution. Final concentrations were 4 nM for both Eu-SA and AF647-SA and 66.6 nM to 4.16 nM for anti-SA. After one hour the TR-FRET values were measured. The second measurement was done after washings. The experiments were performed in duplicate and repeated several times.

In two-step assays 40 µl of polyclonal anti-SA (66.6 nM to 4.16 nM) was added into wells. After 1 hour the wells were washed and the antigen mix (4 nM Eu-SA+4 nM AF647-SA) was added. TR-FRET values were measured after one hour, and after washings. The experiment was performed in duplicate and repeated multiple times.

### Density Gradient Ultracentrifugation

Continous sucrose density gradients were generated as described [24]. Briefly, 0–70% sucrose gradient was generated by layering sucrose solutions (70%, 60%, 50%, 40%, 30%, 20%, 10% and 0%) on top of each other in 14×89 mm Ultra-Clear™ tubes (Beckman Coulter). The gradient was allowed to linearize by keeping the tubes at 4°C overnight prior to centrifugation. Four antigen-antibody combinations were tested: two antigen concentrations for the monoclonal (10 nM AF647-SA+10 nM Eu-SA together with 20 nM yielding a molar ratio of 1∶1:2, and 2.5 nM AF647-SA+2.5 nM Eu-SA together with 20 nM of antibody yielding a molar ratio of 1∶1:8), and two different polyclonal antibodies (20 nM each, Abcam; Springbioscience) with the antigens at 6.6 nM (AF647-SA+Eu-SA) yielding a molar ratio of 3∶1:1 (and approximately 1∶1:1 of specific IgG versus Eu- and AF647-SA). The samples containing the antigen-antibody complexes were separately layered on top of the density gradient and ultracentrifugation (40 000 rpm, 24 h, 5°C) was carried out in SW41 rotor (Beckman Coulter). Fractions of ∼300 µl were collected on a 96-well plate from the bottom of the tube, and the sucrose concentrations were determined using a refractometer.

### Analysis of Protein Complexes in Density Gradient Fractions

Antibodies in the fractions were detected by ELISA. Briefly, 10 µl of each fraction was mixed with 90 µl of 0.1 M Na_2_HCO_3_ (pH 9.3)_,_ pipetted onto 96-well plates and incubated 2 h at 37°C. The plates were blocked with BSA (2 mg/ml in PBS) for 30 min. After blocking, the plates were washed once with PBS-T (PBS+0.05% Tween-20). 100 µl of HRP-labeled secondary (anti-rabbit or anti-mouse) antibody diluted 1 to 1000 in PBS-T was added for 1 h at 37°C. After incubation the wells were washed four times with PBS-T and 100 µl of 3,3′,5,5′-tetramethylbenzidine (TMB) substrate solution (Sigma Aldrich) was added. The reaction was stopped by addition of 50 µl of 0.5 M H_2_SO_4_ and the results were read at 450 nm.

The fractions containing the TR-FRET positive immunocomplexes were determined by pipetting 20 µl of each fraction onto a microplate (ProxiPlate-384 Plus F, Black 384-shallow well Microplate, PerkinElmer) in duplicate, and the TR-FRET values were recorded using Wallac Victor^2^ fluorometer (PerkinElmer).

### Determination of Molecular Weight from Sucrose Concentration

The molecular weights of the protein complexes were determined using precalculated tables [25] for known sucrose concentrations, as described [26].

## Results

### Determination of the Detection Limits for Anti-SA Antibodies in ELISA

**Figure 1:**
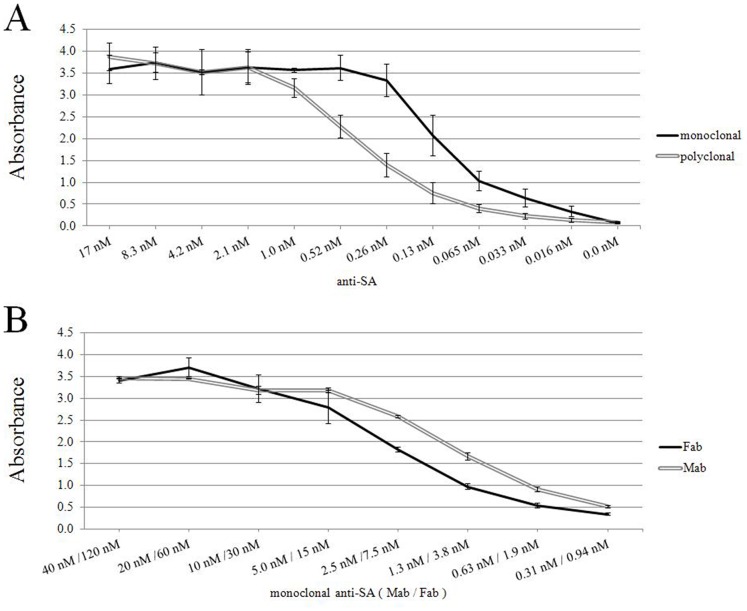
End point titration of the antibodies and testing of Fab fragments. A) Polyclonal antibody versus monoclonal antibody end point titration (17 nM to 0.016 nM) using ELISA. B) A dilution series (0.09 nM to 120 nM) of Fab fragments was tested in SA-ELISA, using monoclonal antibody (0.03 nM to 40 nM) as control. Error bars are ± standard deviation calculated from parallel wells.

We used SA-coated plates to determine the detection limits for the anti-SA monoclonal antibody (MAb) and polyclonal antibody (PAb, rabbit IgG fraction, Abcam Ltd) in ELISA. Additionally, we aimed to get an estimate for the concentration of specific IgG in the IgG fraction of the PAb. The anti-SA antibodies were titrated in twofold steps, in order to determine the highest dilution that produces a signal higher than background. This dilution was found to be 1∶409 600 for MAb and 1∶101 200 for PAb (Fig. 1a). By comparing these end point titers, we conclude that the PAb contains approximately 25–30% of specific IgG. We use this estimate throughout this manuscript and indicate this number in brackets under the whole IgG concentration in figures where applicable.

As an additional control for the downstream assays we prepared Fab fragments from anti-SA MAb by papain cleavage. After cleavage, the Fab fragments were analyzed by SDS-PAGE separation (see Fig. S1). The preparation was shown to be free from intact IgG but did contain trace amounts of Fc parts and inactivated papain. As shown in Fig. 1b the Fab fragments were still capable of binding to SA-coated surface. Subsequently, the optimum dilutions of the anti-SA Fab in relation to the intact anti-SA MAb were assessed from the ELISA result (Fig. 1b). The partially purified Fab fragments were used as control in our TR-FRET assays.

### Antibody-induced TR-FRET of Donor- and Acceptor-labeled Antigens

**Figure 2:**
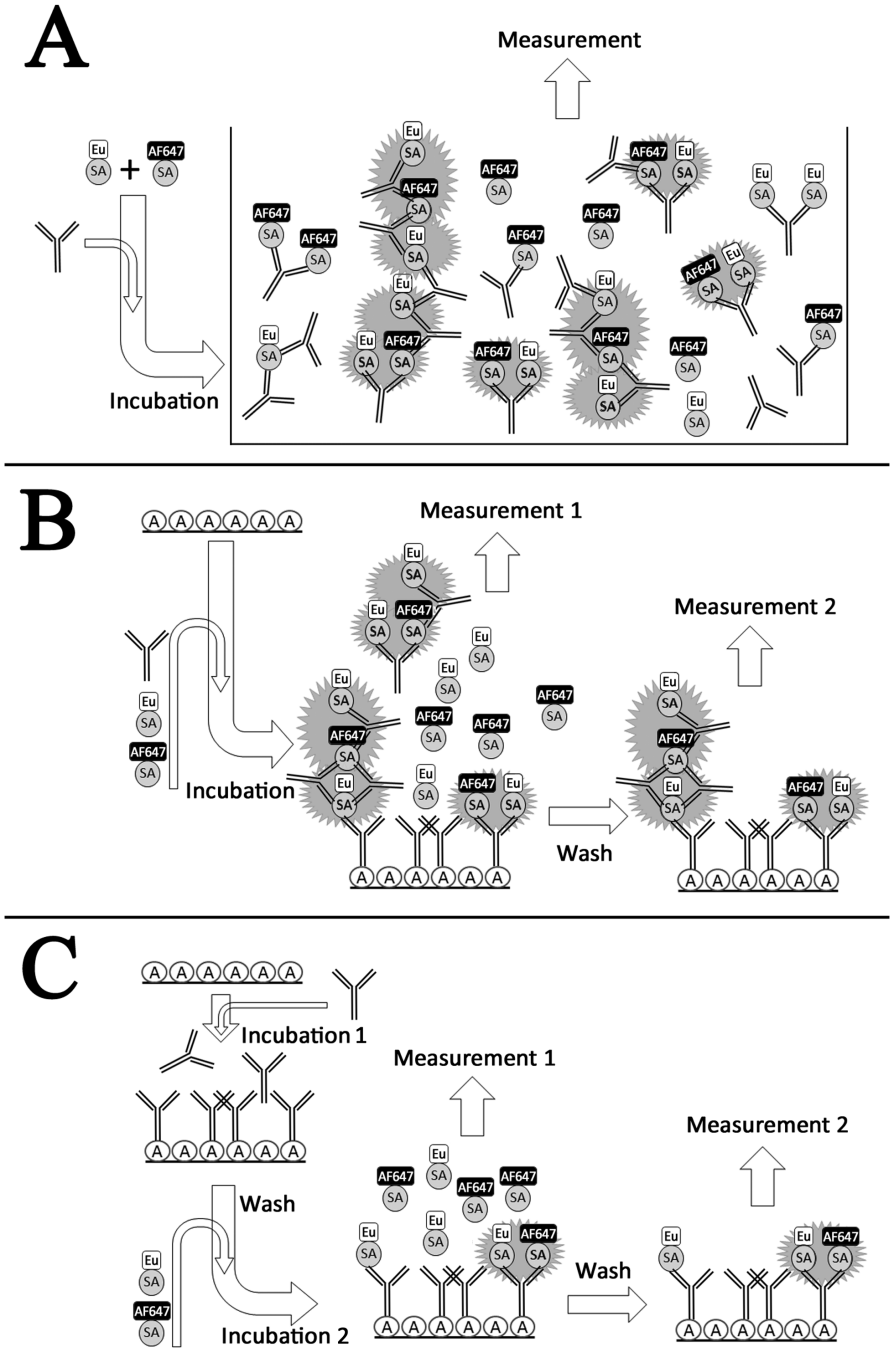
A schematic overview of the experimental procedures. A) Solution-phase assay setup. All reagents were pipetted onto a 384-well plate and the TR-FRET signal read directly. B) One-step solid-phase assay. The antigen mixture and the antibody pipetted onto protein A coated wells and the TR-FRET signal is measured directly (measurement 1) and after replacing the mixture with TBS (measurement 2). C) Two-step solid-phase assay. In the first step the antibody was allowed to bind to a protein A coated well. In the second step equimolar antigen mixture was added. The TR-FRET signal was measured directly after antigen addition (measurement 1) and after replacing the antigen mixture with TBS (measurement 2).

**Figure 3:**
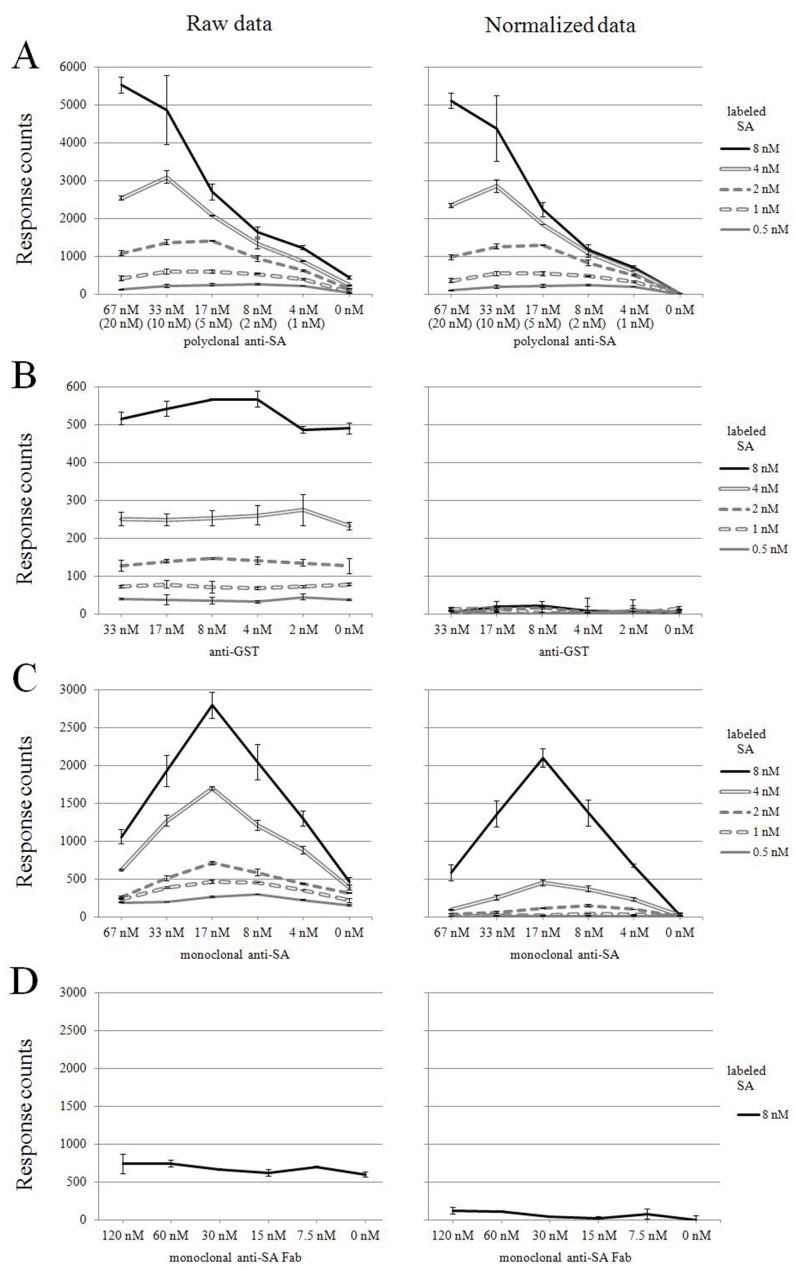
Antibody vs. antigen titration. A) Polyclonal anti-SA antibody (4.16 nM to 66.6 nM), B) polyclonal anti-GST antibody (2.08 nM to 33.3 nM) and C) monoclonal anti-SA antibody (4.16 nM to 66.6 nM) were mixed with five different concentrations of Eu-SA and AF647-SA (at equimolar ratio), and resulting TR-FRET was measured. Dilution series of Fab fragments (7.5 nM to 120 nM) D) tested with 8 nM antigens (Eu-SA and AF647-SA). The TR-FRET (expressed in response counts) is the average of two measurements. Raw data are presented on the left panel, and the normalized data on the right panel. The concentration of specific IgG (for PAb), is indicated in brackets. Error bars are ± standard deviation calculated from parallel wells.

To assess whether an IgG molecule can induce TR-FRET signal via cross-linking donor- and acceptor-labeled antigens, signals induced by antigen-specific vs. unrelated antibodies were compared. Mixtures of Eu- and AF647-labeled SA (in equimolar, serially declining concentrations) were combined with serial dilutions of polyclonal IgG from rabbits immunized either with specific (SA) or control (GST) antigens. Our first experimental set-up was a homogenous, solution-phase assay depicted in Fig. 2A. With the labeled antigens both at 8 nM, the specific IgG induced TR-FRET signal dose-dependently along with antibody concentration (Fig. 3A). At each antibody dilution the TR-FRET signal was proportional to the concentration of the antigen pair. In all, the signal backgrounds in the absence of SA-specific IgG accounted for approximately one tenth of the antibody-induced signals (Fig. 3A). In contrast, the control IgG (anti-GST) showed no dose-dependence (Fig. 3B) in signals that were comparable to the low antibody-free backgrounds of Fig. 3A. Also the monoclonal anti-SA antibody induced dose dependent TR-FRET signals, similarly to the polyclonal anti-SA (Fig. 3C).

Furthermore to confirm that the observed TR-FRET signals indeed are induced by donor- and acceptor-labeled antigens brought into close proximity via bivalent interaction with anti-SA MAb, a set of control experiments were performed using anti-SA Fab fragments. Various amounts of Fab fragments (dilution series from 120 nM to 7.5 nM) were mixed with Eu-SA and AF647-SA (both at 8 nM) and the resulting TR-FRET signals were compared to those induced by intact MAb. As shown in Fig. 3D, the Fab fragments failed to induce TR-FRET signals higher than background, while a typical dose depended signal increase was seen with the intact anti-SA MAb (Fig. 3C).

Moreover, as most of the background is actually not TR-FRET-derived but comes from spillover of Eu fluorescence into the wavelength of measurement (665 nm), we normalized our TR-FRET assay data in this regard (see Materials and Methods). The normalized data are shown in the right-hand panel of Fig. 3, aside of the original (uncorrected) data in the left. As apparent in e.g. Fig. 3B, this elimination of fluorescence spillover brought the background to less than 2% of original, i.e, from ∼500 units (seen with the highest concentration [8 nM] of fluorescent antigens) to <10 units.

Not only the normalized but also the original data of Fig. 3A point to a broad dynamic range of the solution-phase assay; as apparent with the higher concentrations of fluorescent antigens (4 and 8 nM), all dilutions of polyclonal anti-SA IgG emerged distinctly from background. By comparing the end-point dilutions of ELISA and TR-FRET assay (∼1∶400 000 and 1∶1600 for MAb, respectively), we conclude the ELISA to be approximately 250-fold more sensitive than this first generation TR-FRET assay.

### Optimal Ratio of Donor- and Acceptor-labeled Antigens

**Figure 4:**
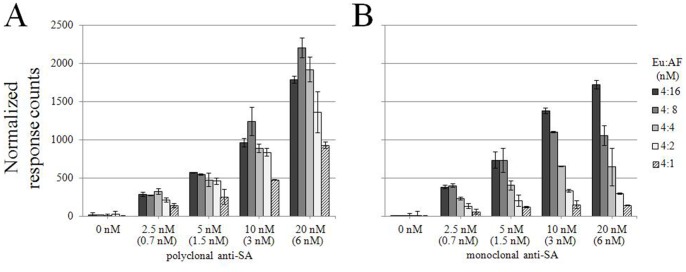
The effect of donor vs. acceptor concentration on the assay performance. A) Polyclonal anti-SA antibody (2.5 nM to 20 nM) was mixed with Eu-SA (constant concentration of 4 nM) and AF647-SA (at concentrations from 1 nM to 16 nM). The TR-FRET values (expressed in normalized response counts) were measured immediately after mixing. B) The same was done with the monoclonal antibody. The concentration of specific IgG (for PAb), is indicated in brackets. Error bars are ± standard deviation calculated from parallel wells.

To determine the optimal ratio of donor- vs. acceptor-labeled antigens, we cross-titrated the polyclonal anti-SA IgG with the Eu-SA and AF647-SA in many different ratios. The antibodies in four dilutions were combined with an antigen mix containing Eu-SA invariably at 4 nM and AF647-SA at 1 to 16 nM. Fig. 4 with normalized TR-FRET signals shows the best signal to noise relation. With PAb the optimum was at or near equimolar ratio of donor- and acceptor-labeled antigens (Eu:AF647 = 4∶8 or 4∶4), while with MAb it was at an 16∶4 excess of the acceptor-labeled antigen. Based on these data we chose to proceed with the donor- and acceptor labeled antigens at equimolar concentrations, as the differences were not remarkable in either way.

### Effect of Incubation Time on Solution-phase Assay Performance

**Figure 5:**
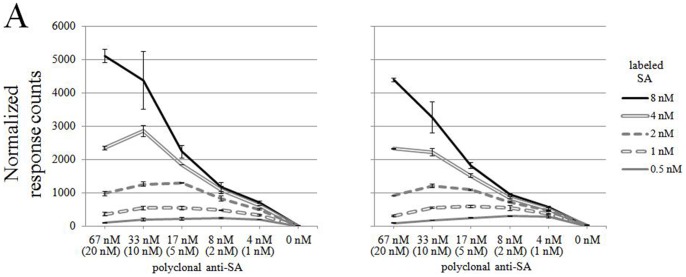
The effect of incubation time on the TR-FRET values. The stability of the formed immune complexes was studied by mixing polyclonal anti-SA antibody (4.16 nM to 66.6 nM) with Eu-SA and AF647-SA (at equimolar ratio from 0.5 nM to 8 mM). The TR-FRET values were measured immediately after mixing the reagents (A) and after 2h incubation at rt (B). The concentration of specific IgG (for PAb), is indicated in brackets. Error bars are ± standard deviation calculated from parallel wells.

Next, we studied the effect of incubation time on the formation of TR-FRET signal. We were also interested in studying the stability of the immune complexes formed in the homogenous assay. The polyclonal IgG was cross-titrated with the fluorescent antigens in a number of ratios, and the signals were measured at several time points (15 min to 24 h) at room temperature (RT) or 4°C. As can be seen in Fig. 5, maximal TR-FRET values were already seen at the earliest time point (15 min) after mixing. Further incubation at 4°C for 2 h or overnight did not alter the TR-FRET values (data not shown); however, at RT a decrease of ∼15% was seen at 2 h that remained unaltered for 24 h (data not shown).

### Effects of Dissociating Agents

**Figure 6:**
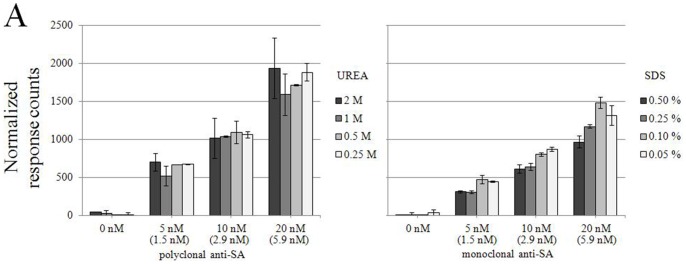
The effect of dissociating agents on immune complex formation. Polyclonal anti-SA antibody (5 nM to 20 nM), Eu-SA and AF647-SA were diluted A) to 0.05–0.5% SDS or B) to 0.25–2 M urea, mixed together and the TR-FRET values (expressed in normalized response counts) were measured. The concentration of specific IgG (for PAb), is indicated in brackets. Error bars are ± standard deviation calculated from parallel wells.

As immunoassays are often performed in the presence of protein denaturants or detergents in low concentrations, to diminish background, we tested the susceptibility of the homogenous assay to urea and SDS. Again, the antibody in various concentrations was mixed with the fluorescent antigens in the presence of low concentrations of the two agents. As illustrated in Fig. 6, urea at up to 2 M had little effect on the TR-FRET signal, which was somewhat inhibited by SDS at 0.5%, yet not much at 0.25%.

### Isolation and Characterization of the FRET-inducing Complex

To estimate the sizes of the complexes responsible for the TR-FRET signal, we separated them by sucrose gradient ultracentrifugation. Both the polyclonal and monoclonal anti-SA antibodies were employed at 20 nM. With the former the fluorescent antigens were used at 6.6 nM, and with the latter at 2.5 or 10 nM. The mixtures were separately ultracentrifuged, and the fractions analyzed for TR-FRET activity and antibody as well as sucrose concentration.

**Figure 7:**
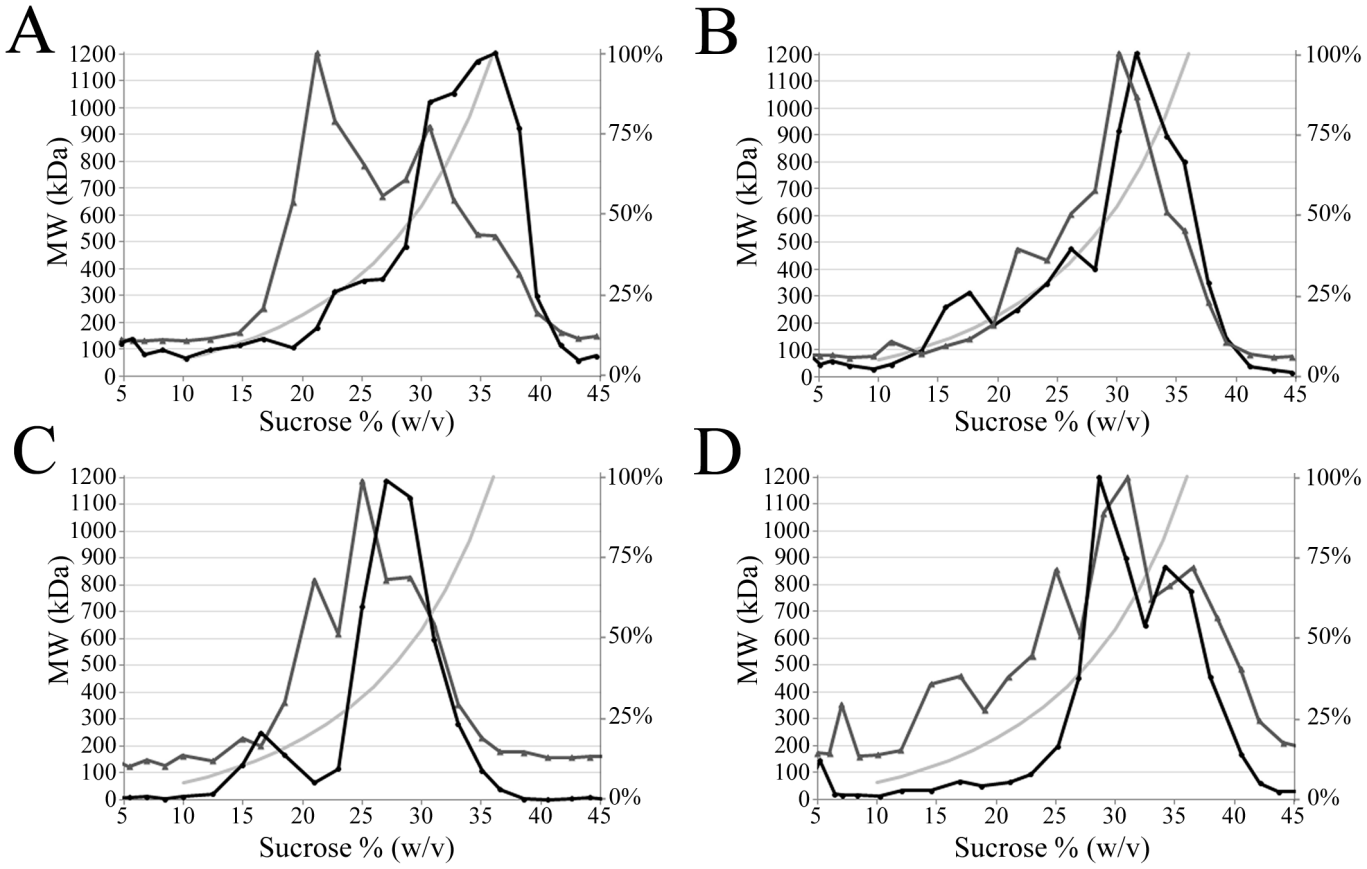
Size determination of immune complexes in density gradient ultracentrifugation. Immune complexes were formed by mixing A) Monoclonal anti-SA, Eu-SA and AF647-SA at 8∶1:1 ratio B) Monoclonal anti-SA, Eu-SA and AF647-SA at 2∶1:1 ratio C) Polyclonal anti-SA (Abcam), Eu-SA and AF647-SA at 3∶1:1 ratio D) Polyclonal anti-SA (Springbioscience) at 3∶1:1 ratio, and separated by ultracentrifugation in preformed 0–70% sucrose gradient. Fractions collected from bottom were analyzed for TR-FRET activity, sucrose concentration and antibody concentration. The black line represents relative TR-FRET value (each value divided by the highest measured value) and the dark grey line represents relative antibody concentration (normalized to the highest value as above) in each fraction. Estimated molecular weight in kilodaltons (Y-axis) is calculated according to each sucrose concentration (X-axis) and shown in light grey line. The second y-axis is a relative scale (from 0–100% of the TR-FRET in fraction with highest TR-FRET signal) used for both TR-FRET signal and antibody amount.

As shown in Fig. 7A–B, most of the FRET activity sedimented in high-density fractions corresponding to relative molecular mass (Mr) of 500 to >1000 kDa, when using MAb. In Fig. 7A (antibody to antigen ratio 4∶1) most of the MAb migrates in fractions that are not TR-FRET positive. On the other hand in Fig. 7B (antibody to antigen ratio 1∶1) most of the MAb migrates to fractions that are TR-FRET positive. Thus the proportion of MAb brought into these (TR-FRET-positive) high-density fractions depended on the antigen to antibody ratio in the original MAb-SA mixture (compare Fig. 7A to B). Furthermore, the results suggest that the MAb to antigen ratio of 1∶1 is close to optimal, since virtually no free MAb was observed. Minor proportion of FRET signal was observed in lower-density fractions corresponding to Mr of ∼300 kDa.

Figs. 7C–D show the corresponding results using polyclonal antibodies from two commercial sources. Interestingly, the TR-FRET positive complexes generated by the two differed somewhat in size. While the peak fractions in Fig. 7C correspond to M_r_ of 350 to 700 kDa, those in Fig. 7D resemble in size those obtained using MAb (Fig. 7A–B). This difference between the two immunoglobulin preparations may be due to a different proportion of SA-specific antibody present in the two. Altogether, in the homogenous solution-phase assay, most of TR-FRET signal originated from immune complexes greater in size than a single IgG+Eu-SA+AF647-SA (160 kDa+60 kDa+60 kDa = 280 kDa). However, a minor proportion of the signal came from immune complexes of relatively small size range.

### Two-step Solid-phase Assay

**Figure 8:**
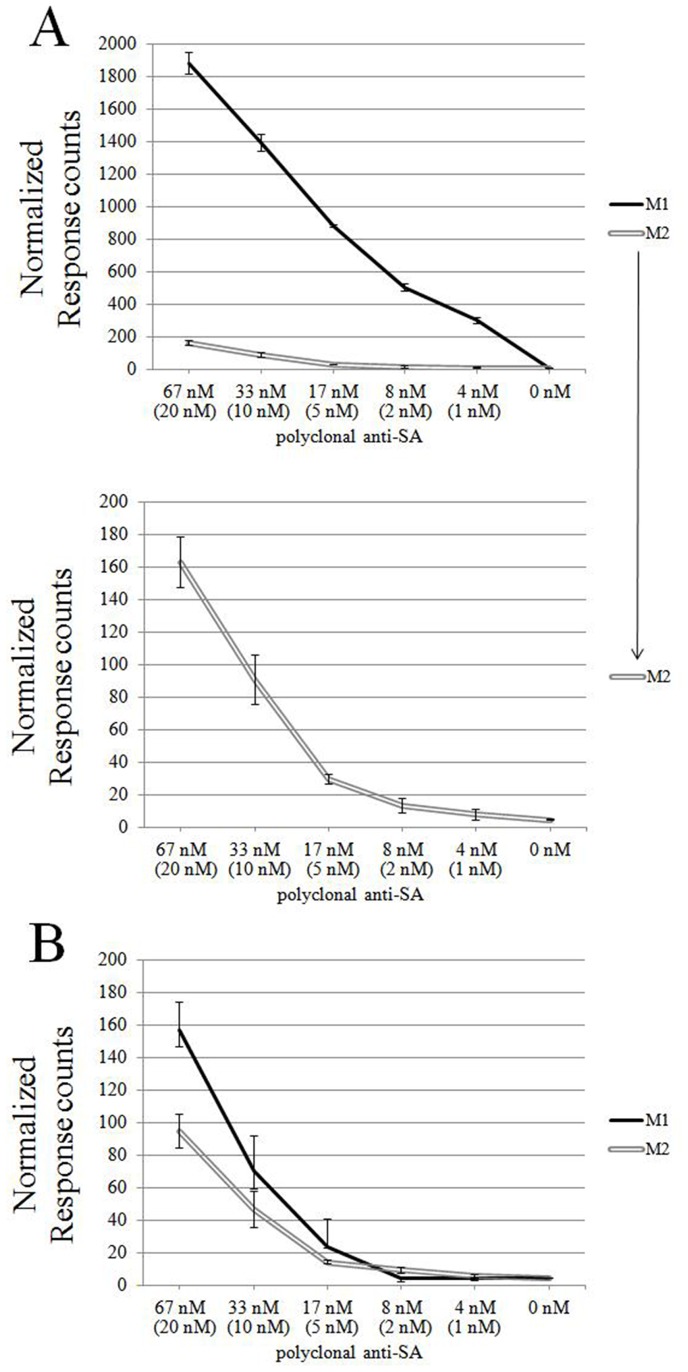
Two-step and one-step solid-phase TR-FRET assays. A) Polyclonal anti-SA antibody (at 66.6 nM to 4.16 nM) was pipetted with antigen mixture (Eu-SA and AF647-SA; 4 nM) onto microwells coated with protein A. The TR-FRET was measured directly after incubation (M1) and after replacing the reaction solution with TBS (M2). The M2 values are also represented separately. B) Polyclonal anti-SA antibody (66.6 nM to 4.16 nM) was immobilized via protein A onto a 384-well plate and excess antibodies were removed prior to adding the antigen mixture (Eu-SA and AF647-SA, both at 4 nM final concentration). TR-FRET was measured directly after incubation (M1) and after replacing the antigen mixture with TBS (M2). The concentration of specific IgG (for PAb), is indicated in brackets. Error bars are ± standard deviation calculated from parallel wells.

To further investigate the nature of FRET-pair formation, we set up an assay wherein the formation of larger immune complexes is prevented. Polyclonal anti-SA IgG (66.6 nM to 4.16 nM) was immobilized on microwells via protein A (Fig. 2C). After removal of unbound antibodies the labeled antigens (Eu-SA and AF647-SA; at 4 nM) were added, and TR-FRET was measured both before and after removal of unbound antigens (M1 and M2 in Fig. 8B). The TR-FRET signals were generated, in synchrony with the IgG concentration, as in the solution-phase assay (Fig. 3), albeit at a much lower level. Removing unbound and weakly bound antigens lowered the signal considerably.

### Single-step Solid-phase Assay

To further assess the performance characteristics of the solution-phase and solid-phase assays, the polyclonal anti-SA (66.6 nM to 4.16 nM) and the labeled antigens (Eu-SA and AF647-SA; 4 nM) were pipetted simultaneously onto protein A-coated microwells (Fig. 2B). As in the two-step solid-phase assay, the TR-FRET values were measured both before and after removal of unbound IgG and antigens. While most of the signal was lost upon removing unbound antigen-antibody complexes (M1 and M2 in Fig. 8A), those remaining immobilized by protein A accounted for similar TR-FRET values as two-step assay before unbound antigen removal (Fig. 8B M1).While the signal levels were fairly low, the background without specific antibody was only of a few counts, achieving high signal to noise ratios with both solid phase assays.

## Discussion

In this report we describe a novel TR-FRET based approach for detection of antibodies from biological fluids using a solution-phase homogenous assay and solid-phase heterogenous assays. We demonstrated with donor- and acceptor-labeled SA that addition of a specific antibody to a mixture of antigens each carrying one of the two labels induces the formation of immune complexes producing a TR-FRET signal promptly after combining the reagents. Once formed the immune complexes appeared stable, and even 24 h storage at 4°C did not markedly alter the TR-FRET values. By antigen-antibody cross-titrations we observed the assays to be applicable over a wide range of IgG concentrations. We also studied the effects of dissociating agents on the immune complex formation. Size-estimation by density gradient ultracentrifugation showed that most of the TR-FRET signal is derived from large immune complexes (mw >500 kDa), while some signal also emerged from smaller complexes (mw 250–400 kDa). Finally, we set up a protein A based solid-phase assay to further investigate the TR-FRET assay we have established.

We initially compared the sensitivity of detection of the TR-FRET assay with that of ELISA. Furthermore, we used the ELISA result to obtain an estimate of the amount of specific IgG in the PAb. Even though the exact concentration of specific IgG molecules cannot be told through this assay, the result (25–30% of specific IgG in the PAb) correlate with our TR-FRET assay data in that, the maximum TR-FRET signal (peak) with PAb comes at 2 to 4 times higher antibody concentration than with MAb, compare A and C in Fig. 3. The endpoint titration experiments in ELISA also gave us the opportunity to compare the TR-FRET assay with a conventional immunoassay. Obviously, the analytical sensitivity of ELISA was higher (about 250 times) than that of the TR-FRET assay, most likely due to the enzymatic reaction inherent in the former. However, we feel the sensitivity of the TR-FRET application to be sufficient for the assay to be applicable to serodiagnostic use.

While varying the ratios of antigen to antibody, we observed the TR-FRET values to increase in relation to the specific antibody (mono- or polyclonal) concentrations. Yet, we observed that beyond a peak (TR-FRET) signal, the values started to decline. The most obvious explanation is a prozone effect in which only one of the IgG molecule’s Fab arms attaches to an antigen, leaving the other empty. Based on our original hypothesis (in which the donor and acceptor are brought to close proximity via specific IgG), the ideal molar ratio of the antigens and antibody could be assumed to be 1∶1:1 (donor:acceptor:IgG). However, the phenomenon in practise seems more complex.

According to the ultracentrifugation experiments (Fig. 7A to D), the TR-FRET activity mostly derives from immune complexes of various sizes, with both MAb and PAbs. With monoclonal anti-SA, most of the TR-FRET signal originated from immune complexes >700 kDa. Complexes of this size could include e.g. three IgG molecules (ca. 450 kDa) and four antigen molecules (ca. 250 kDa). There was also some TR-FRET signal in density gradient fractions that by molecular weight correspond to an immune complex potentially containing one molecule of IgG and donor-labeled and acceptor-labeled antigens. In all, it seems likely that most of the TR-FRET signal does come from complexes larger than one IgG plus two antigen molecules, as also suggested by the solid-phase TR-FRET assay.

One point that needs to be taken into account is that the antigen (SA) used in our experiments is a tetravalent molecule. One reason why we initially chose this antigen is that it is commercially available as both donor- and acceptor-fluorophore conjugates. The tetrameric nature of SA in theory permits the attachment of four IgG molecules (ca. 600–700 kDa) to one SA molecule (in the case of a MAb). On the other hand, only steric considerations will restrict the number of IgG molecules that potentially binds to a single SA molecule. Thus to prove our initial hypothesis, one would need a monovalent antigen and a monoclonal antibody. In fact, the PAb in our TR-FRET assay actually yielded much higher fluorescense counts than the MAb. The use of PAb seems to favor the formation of bulky immune complexes responsible for TR-FRET signals via non-covalent cross-linking of antigen molecules. Since PAb recognizes a multiplicity of epitopes, it might allow formation of tighter antigen-antibody complexes than a MAb thus further explaining the higher TR-FRET values.

With the two-step solid-phase assay we further investigated the nature of FRET-pair formation. By immobilizing anti-SA with protein A and removing any unbound antibodies before the addition of labeled antigens we prohibited the formation of larger immune aggregates. A polyclonal IgG fraction usually contains only a small portion (5–10%) of IgG molecules specific to the antigen. Protein A, on the other hand, binds all IgGs regardless of antigen specifity. Thus in the protein A bound antibody population these specific antibodies very likely are too far from each other to allow inter-antibody FRET-pair formation. Unfortunately the polyclonal anti-SA we used seems to contain far too many SA-specific antibodies for this hypothesis to hold true. In future experiments it could be tested whether the low sensitivity of this solid phase assay allows diluting our anti-SA with another antibody, such as anti-GST, to significantly lower the portion of SA-specific IgGs. These results together with the single-step solid-phase assay show that TR-FRET –based applications are viable in many different setups.

Liu *et al*. [9] introduced a homologous immunoassay based on two-photon excitation fluorescence resonance energy-transfer (TPE-FRET). They detected polyclonal anti-BSA antibodies using differentially labeled BSA molecules. Their results are in line with ours on SA and anti-SA, although the techniques are somewhat different. We also verified the phenomenon using a monoclonal antibody. It is not impossible that also their results are based on larger immune complexes, as they did not assess in detail the molecular determinants of their findings. It should nevertheless be taken into account that BSA is a monomer at the concentrations used in the study implying that the FRET-bridge assay should be applicable with monomeric antigens. On the other hand, we wish to pinpoint that most of the microbial antigens do naturally exist as multimers (di- or trimers or larger).

The homogenous TR-FRET immunoassay set up herein is rapid and robust, and flexible in terms of incubation time and temperature. After this demonstration of proof-of-concept, in the future we plan to label diagnostic antigens with donor and acceptor fluorophores, and to experimentally demonstrate that the assay is applicable to wash-free human serodiagnostics. Notably, the average concentrations of IgG antibodies detected in human sera (∼4–16 mg/ml of which antibody of particular specificity comprises only a few percent) are even higher than those used in our experiments. The solution-phase TR-FRET based immunoassay principle presented herein has potential to emerge as a major approach in diagnostic antibody detection.

## Supporting Information

Figure S1 **Fragmentation of anti-streptavidin MAb.** The lanes in SDS-PAGE are: 1. marker (Precision Plus Protein™ Dual Color Standards, Bio-Rad), 2. blank, 3. Gammabind beads (the Fc parts), 4. concentrated Fab fragments, 5. unused Gammabind beads, 6. intact anti-streptavidin MAb. The molecular mass markers (visible on gel) are 250 kDa, 150 kDa, 100 kDa, 75 kDa, 50 kDa and 37 kDa by size.
(TIF)
