# Supplementary figures and images for: Time-Resolved FRET -Based Approach for Antibody Detection – A New Serodiagnostic Concept
Source: PLoS One. 2013 May 7;8(5):e62739. doi: 10.1371/journal.pone.0062739 (PMC3647052; doi:10.1371/journal.pone.0062739)

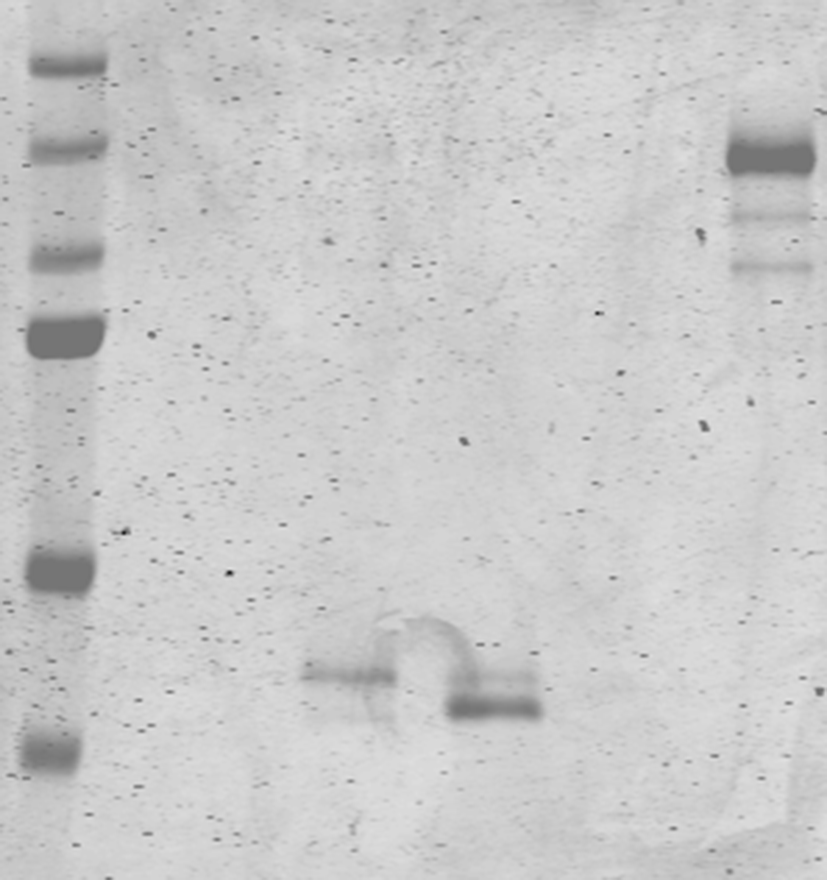

Supplement: Figure S1 — Fragmentation of anti-streptavidin MAb. The lanes in SDS-PAGE are: 1. marker (Precision Plus Protein™ Dual Color Standards, Bio-Rad), 2. blank, 3. Gammabind beads (the Fc parts), 4. concentrated Fab fragments, 5. unused Gammabind beads, 6. intact anti-streptavidin MAb. The molecular mass markers (visible on gel) are 250 kDa, 150 kDa, 100 kDa, 75 kDa, 50 kDa and 37 kDa by size. (TIF) [file pone.0062739.s001.tif]
